# Case Report: Ultrasound-guided nerve mobilization and rehabilitation for non-traumatic posterior interosseous nerve palsy associated with a ganglion cyst

**DOI:** 10.3389/fresc.2026.1811498

**Published:** 2026-05-20

**Authors:** Tomonori Hayashi, Katsuyuki Morishita, Kazuhiko Minagawa

**Affiliations:** 1 Faculty of Arts and Sciences at Fujiyoshida, Showa Medical University, Yamanashi, Japan; 2 Minagawa Orthopaedic Clinic, Tokyo, Japan; 3 Department of Rehabilitation Science, Graduate School of Health Sciences, Josai International University, Chiba, Japan

**Keywords:** ganglion cyst, peripheral nerve disorder, physical therapy, posterior interosseous nerve palsy, radial nerve entrapment, rehabilitation, ultrasound-guided nerve mobilization

## Abstract

Nontraumatic posterior interosseous nerve (PIN) palsy is an uncommon entrapment neuropathy, and there is limited evidence regarding ultrasound-guided nerve mobilization as part of rehabilitation. Here, we report a case of PIN palsy associated with a ganglion cyst in the radial tunnel, focusing on the clinical course and ultrasound-based evaluation of nerve morphology and motion. The patient presented with dorsal forearm pain, marked weakness of the fingers, and thumb extension. Ultrasonography revealed a hypoechoic ganglion compressing and flattening the PIN near the arcade of Frohse. The initial management consisted of weekly ultrasound-guided aspiration of the ganglion combined with conventional physical therapy, including low-frequency electrical stimulation, stretching, and active-assist exercises for finger extension. Although pain decreased promptly after decompression, motor recovery remained limited, and follow-up ultrasonography showed persistent nerve flattening and reduced longitudinal gliding. Therefore, an ultrasound-guided nerve mobilization program was added to restore nerve gliding around the compression site while continuously monitoring the nerve and its surrounding structures. Over subsequent sessions, finger extension strength and hand function improved in parallel with a reduction in the nerve cross-sectional area, normalization of the flattening ratio, and qualitative recovery of nerve sliding on ultrasonography. This case suggests that ultrasound-guided nerve mobilization may be a useful adjunct to decompression and exercise therapy in PIN palsy related to ganglion compression and highlights the value of serial ultrasound assessment, including quantitative evaluation such as particle image velocimetry (PIV), for guiding and validating rehabilitation in peripheral nerve disorders.

## 1 Introduction

Posterior interosseous nerve (PIN) palsy is an uncommon entrapment neuropathy of the deep branch of the radial nerve, typically presenting with weakness of the finger and thumb extension without cutaneous sensory loss (1, 2). This condition arises within the radial tunnel, where several anatomical structures, including the fibrous proximal margin of the supinator, known as the arcade of Frohse, predispose the nerve to compression (3, 4). The arcade of Frohse is present in approximately 66% of adult forearms and is the most frequent site of entrapment (3). In addition to anatomical narrowing, space-occupying lesions such as ganglion cysts or lipomas may also compress the PIN within the radial tunnel (2). The clinical presentation of PIN palsy often overlaps with other causes of lateral elbow pain and extensor weakness, making the diagnosis challenging (5–7). Furthermore, the incidence of radial tunnel syndrome and compressive PIN neuropathy is relatively low compared to that of other upper-extremity entrapment neuropathies (5, 6), contributing to delays or misclassification in clinical practice.

High-resolution ultrasonography enables real-time visualization of nerve morphology, dynamic assessment of excursion, and guidance for minimally invasive procedures in entrapment neuropathies (8–10). However, there is limited evidence on how serial ultrasound assessments can be integrated with rehabilitation strategies to monitor nerve recovery and guide nerve mobilization techniques in patients with PIN palsy (10). Furthermore, objective evaluation of nerve gliding using techniques such as particle image velocimetry (PIV) has rarely been applied to validate these interventions *in vivo*. We encountered a patient with nontraumatic PIN palsy associated with a ganglion cyst in the radial tunnel who presented with dorsal forearm pain and marked weakness of the finger and thumb extension without sensory disturbance. Although the pain improved after ultrasound-guided aspiration of the ganglion, motor recovery remained limited. Follow-up ultrasonography revealed persistent nerve flattening and reduced gliding at the compression site, prompting us to implement an ultrasound-guided nerve mobilization program in addition to conventional physical therapy.

## 2 Case description

A right-handed female cook in her 40s presented with a 2-week history of worsening dorsal forearm pain. Symptoms developed without trauma. She reported inability to fully extend her fingers and difficulty with daily grasping tasks. She did not notice any numbness or tingling of the forearm or hands. Physical examination revealed no visible swelling or deformity in the right elbow. Manual muscle testing revealed marked weakness of the finger and thumb extensions innervated by the PIN, whereas wrist extension with radial deviation was relatively preserved. The sensation over the dorsum of the hand and forearm was intact, and Tinel's sign over the radial tunnel was negative. At the initial visit, her pain intensity on a 0–10 numerical rating scale (NRS) was 4/10. Upper extremity disability was moderate to severe, with a QuickDASH score of 54.5/100 and a HAND20 score of 81/100. QuickDASH is an 11-item patient-reported questionnaire that assesses upper extremity disability and symptoms, with higher scores indicating greater disability (11). The HAND20 is a 20-item, region-specific outcome measure widely used in Japan to evaluate functional impairment of the hand and upper extremity on a scale of 0–100, with higher scores indicating worse function (12).

**Figure 1:**
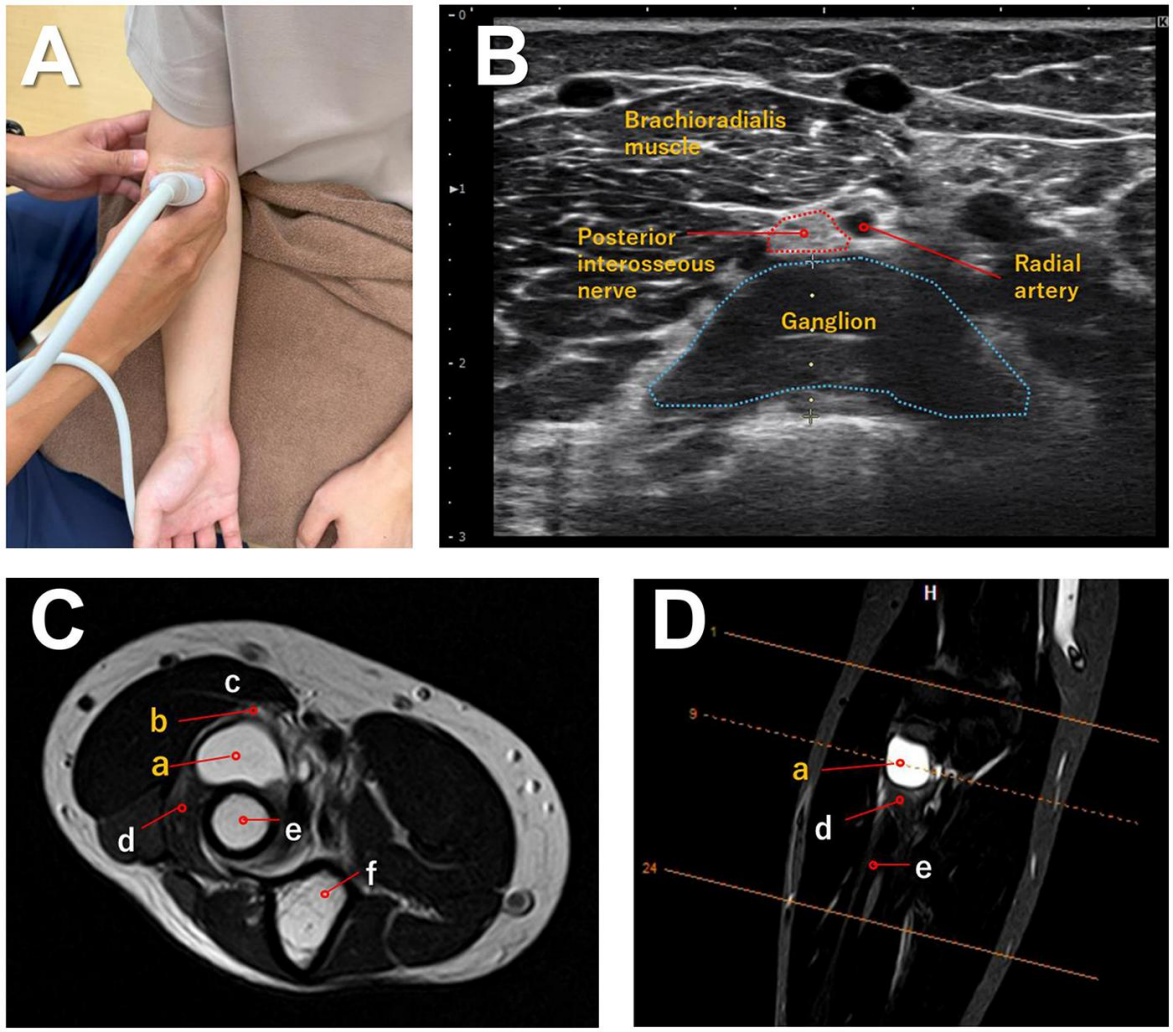
Ultrasound and magnetic resonance imaging of posterior interosseous nerve compression at the proximal forearm. **(A)** Photograph showing the probe placed over the proximal dorsal forearm with the elbow flexed and the forearm supinated. **(B)** Short-axis ultrasound image demonstrating the posterior interosseous nerve compressed between the brachioradialis muscle and a large ganglion in the radial tunnel, adjacent to the accompanying vessels. The cross-sectional areas of the nerve and ganglion are outlined, and the posterior interosseous nerve shows a flattened transverse shape compared with the contralateral side. **(C)** Axial T2-weighted magnetic resonance image at the same level showing the posterior interosseous nerve **(a)** compressed by a hyperintense ganglion **(b)** between the brachioradialis muscle **(c)** and the supinator muscle **(d)** The radius **(e)** and ulna **(f)** are also indicated. **(D)** Coronal T2-weighted magnetic resonance image demonstrating the ganglion **(a)** along the course of the posterior interosseous nerve, with the supinator muscle **(d)** and radius **(e)** labeled; the dashed line indicates the level of the axial slice shown in **(C).**

Routine laboratory test results were within normal limits, and plain radiographs of the elbow showed no bony abnormalities. High-resolution ultrasonography of the radial tunnel revealed a hypoechoic ganglion cyst near the arcade of Frohse at the proximal portion of the supinator muscle. The ganglion compressed the PIN at this level, where the nerve appeared flattened with a qualitative enlargement of its cross-sectional area and blurring of the normal fascicular pattern, consistent with entrapment neuropathy associated with ganglion cysts (13) (Figures 1A,B).

## 3 Diagnostic assessment, therapeutic interventions, and outcomes

Electrophysiological studies, including nerve conduction studies and electromyography, were not performed in this case due to the unavailability of the required equipment at our clinic, which constitutes a limitation of the diagnostic workup. However, the combination of clinical findings—painless wrist extension with radial deviation, and marked weakness of finger and thumb extension without sensory loss—and clear structural changes on high-resolution ultrasonography was highly consistent with PIN palsy. Furthermore, magnetic resonance imaging (MRI) of the elbow performed at another hospital clearly demonstrated a cystic mass with high signal intensity on T2-weighted images of the radial tunnel, compatible with a ganglion compressing the PIN (Figures 1C,D). Given these definitive non-invasive findings, we opted to proceed promptly with conservative management without referring the patient for invasive testing.

At our clinic, high-resolution ultrasonography is performed by physicians for diagnosis and assessment of therapeutic interventions. At the initial visit, a physician-performed ultrasound examination revealed a hypoechoic ganglion located near the arcade of Frohse at the proximal portion of the supinator muscle, compressing and flattening the PIN. The nerve at the compression site had a cross-sectional area (CSA) of 1.72 mm^2^ with a flattened contour and blurring of the normal fascicular pattern, while the ganglion CSA was 67.06 mm^2^. Based on these findings, we initiated a treatment program consisting of ultrasound-guided aspiration of the ganglion combined with conventional physical therapy.

**Figure 2:**
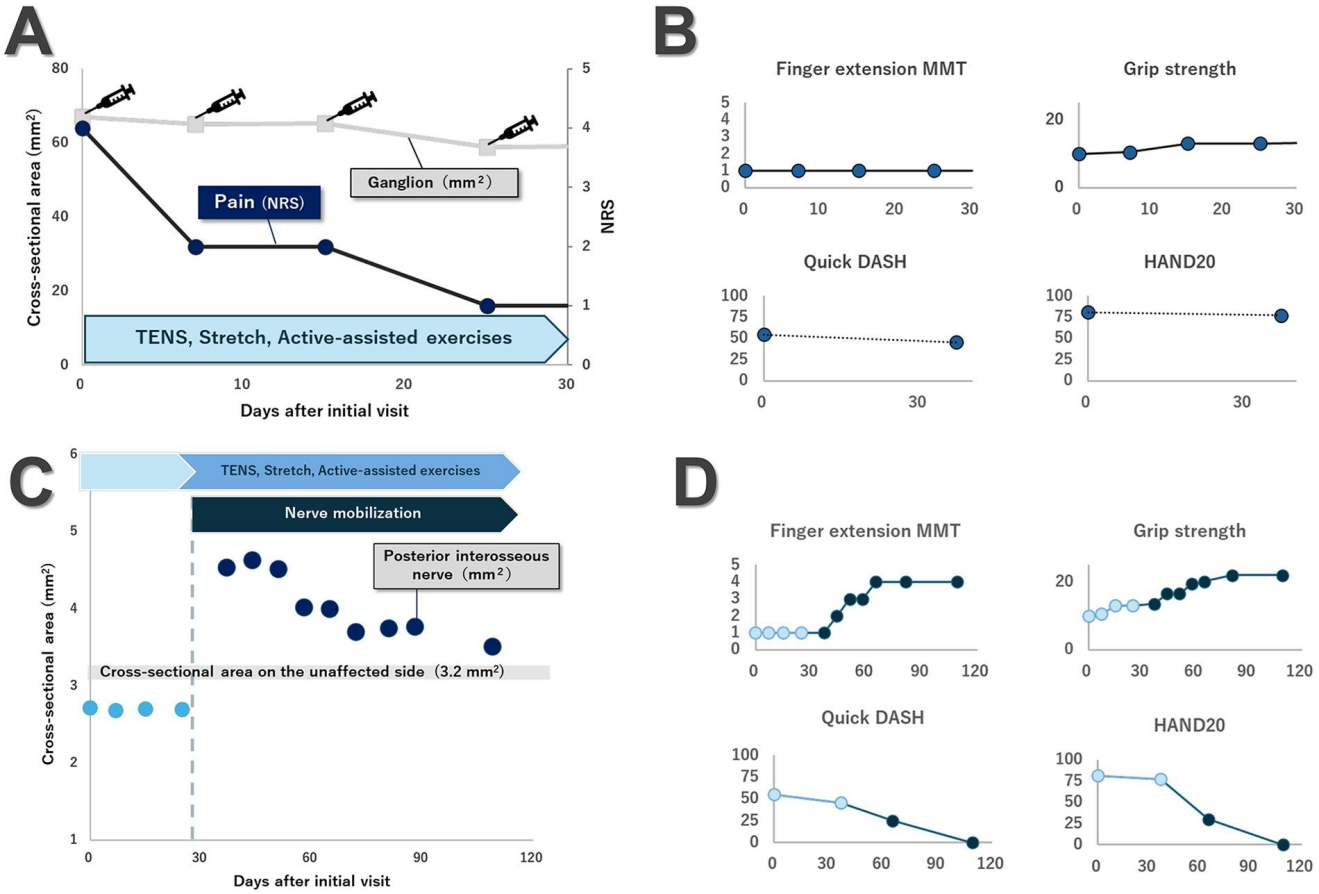
Treatment program and clinical course before and after the introduction of nerve mobilization. **(A)** Time course of ganglion cross-sectional area (CSA) and pain intensity during the first 4 weeks after the initial visit. The patient received once-weekly ultrasound-guided aspiration in combination with a conventional physical therapy program consisting of low-frequency TENS, stretching, and active-assisted exercises (two 20-minute units per session). With each aspiration session, the ganglion CSA gradually decreased and pain on the numerical rating scale (NRS) improved from 4/10 to 1/10. **(B)** Changes in finger extension MMT, grip strength, QuickDASH, and HAND20 scores over the same initial 4-week period. Despite the reduction in pain and ganglion size, improvements in motor function and upper-limb disability remained limited at 4 weeks.**(C)** Time course of the cross-sectional area of the posterior interosseous nerve at the compression site from the initial visit to day 109. The shaded horizontal band represents the CSA of the unaffected side (3.2 mm^2^). The initial program of TENS, stretching, and active-assisted exercises was continued, and ultrasound-guided nerve mobilization was added from day 30 onward (vertical dashed line). After the start of nerve mobilization, nerve CSA increased transiently and then gradually decreased toward the contralateral range during follow-up. **(D)** Changes in finger extension MMT, grip strength, QuickDASH, and HAND20 scores from the initial visit to day 109. The vertical dashed line again indicates the introduction of nerve mobilization. Following the addition of nerve mobilization, motor and functional outcomes—previously showing only limited improvement—began to improve steadily, and rehabilitation was completed on day 109, when pain had resolved and upper-limb function had almost fully recovered.

During the initial 4-week aspiration phase, ultrasound-guided aspiration was performed once weekly (four sessions total), and physical therapy was provided once weekly (two 20-min units). The program included low-frequency transcutaneous electrical nerve stimulation (TENS) to the dorsal forearm (80–100 Hz, sensory-level intensity), static stretching of the flexor muscles, and active-assisted finger extension exercises using the tenodesis effect. During this period, dorsal forearm pain decreased substantially from a NRS of 4/10 to 1/10; however, finger and thumb extension strength remained poor [Manual Muscle Testing (MMT) grade 1], and grip strength increased only modestly from 10 to 13 kg. Serial ultrasound demonstrated a reduction in ganglion CSA to 58.96 mm^2^, yet the nerve CSA remained flattened at approximately 1.70 mm^2^ (Figures 2A,B; Table 1). Crucially, dynamic scanning in the frontal plane confirmed persistent sliding dysfunction, with almost no visible relative motion between the PIN and the overlying brachioradialis muscle.

**Table 1:** Serial changes in imaging findings and clinical outcome measures from the initial visit to the final follow-up.

Days after initial visit	Ganglion CSA (mm^2^)	Posterior interosseous nerve CSA (mm^2^)	Pain (NRS)	Finger extension MMT	Right Grip strength (kg)	Quick DASH	HAND20
0	67.06	1.72	4	1	10	54.5	81
7	65.14	1.68	2	1	10.5		
15	65.255	1.70	2	1	13		
25	58.96	1.69	1	1	13		
37	59.17	3.54	1	1	13.5	45.5	77
44	53.11	3.63	0	2	16.5		
51	53.25	3.51	0	3	16.5		
58	53.91	3.02	0	3	19.4		
65	46.42	2.99	0	4	20.1		
72	40.81	2.70	0				
81	41.145	2.75	0	4	22		
88	37.41	2.77	0				
109	37.13	2.51	0	4	22	0	0

CSA, cross-sectional area; NRS, numerical rating scale; MMT, manual muscle testing.

To ensure measurement reliability, the cross-sectional areas of both the ganglion and the PIN were measured three times by the same examiner on stored ultrasound images. Intra-rater reliability was excellent, with ICC (3,1) values of 0.93 (95% CI: 0.86–0.96) for the PIN and 0.88 (95% CI: 0.75–0.94) for the ganglion measurements.

Consequently, approximately 4 weeks after the initial visit, we modified the rehabilitation program by adding ultrasound-guided nerve mobilization to address this persistent sliding impairment. During the first session, the nerve was visualized in the frontal plane, and gentle manual mobilization was applied with the elbow in flexion and the forearm in supination to promote longitudinal sliding. The therapist maintained a verbal rhythm of approximately 2 Hz to provide consistent pacing. The mobilization procedure lasted approximately 10 min per session. Each set consisted of 20 to 30 repetitions at the aforementioned 2 Hz rhythm, and multiple sets were performed. During the procedure, while maintaining a short-axis view, the ultrasound probe was dynamically shifted proximally, distally, and transversely to specifically target and mobilize multiple sites demonstrating restricted nerve gliding. The applied manual force was strictly adjusted to ensure the patient experienced no pain, with continuous verbal feedback confirming a pain-free state. Under real-time monitoring, movement of the superficial soft tissues over the nerve became apparent, indicating qualitative improvement in sliding. To objectively validate these observations, we performed exploratory PIV analysis (Flow-PIV, Library Co., Ltd.) using ultrasound videos (30 frames/s; SONIMAGE SNiBLE 2, Konica Minolta) acquired immediately before and after this first session. Statistical analysis (Welch's *t*-test, R software) demonstrated a significant improvement in the mean tissue gliding velocity, increasing from 0.35 ± 0.18 cm/s to 0.50 ± 0.28 cm/s (p = 0.047, Cohen's d = 0.60).

**Figure 3:**
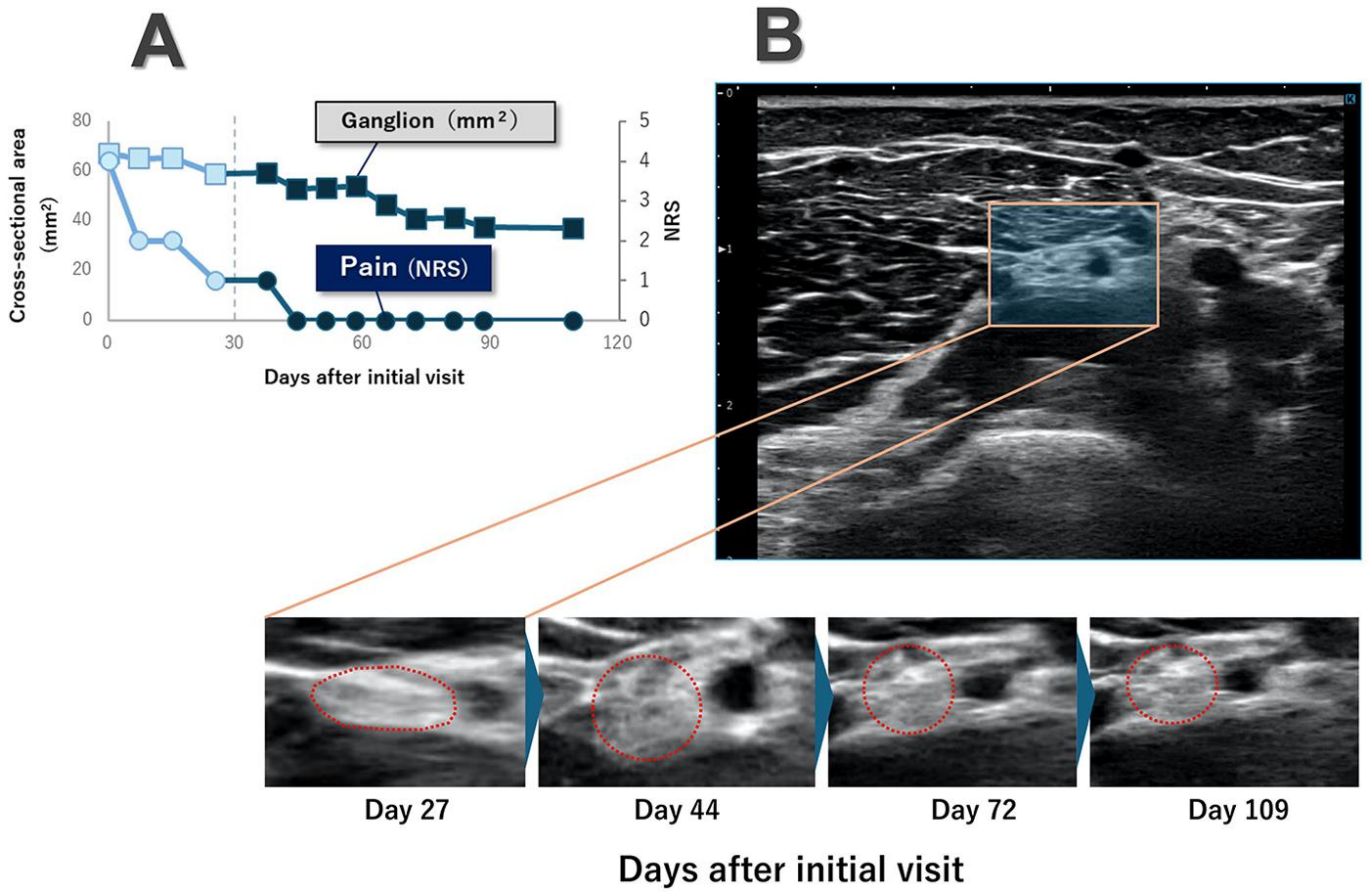
Time course of ganglion size and pain and serial short-axis ultrasound images at the compression site. **(A)** Time course of ganglion cross-sectional area (CSA) and pain intensity from the initial visit to day 109. The patient initially received once-weekly ultrasound-guided aspiration combined with a conventional physical therapy program. Ultrasound-guided nerve mobilization was added from around day 30 onward (vertical dashed line). After the introduction of nerve mobilization, ganglion CSA and pain on the numerical rating scale (NRS) decreased further and then stabilized at low levels during follow-up. **(B)** Short-axis ultrasound images of the posterior interosseous nerve and its accompanying vessel at identical magnification obtained at days 27, 44, 72, and 109 after the initial visit. The posterior interosseous nerve is outlined by a red dotted line, and the accompanying vessel is visible to its right. On day 27 (before the introduction of nerve mobilization), the nerve and vessel appear flattened. After the initiation of ultrasound-guided nerve mobilization (day 44), both structures show an increase in vertical diameter with a more rounded profile, followed by a gradual reduction in size on days 72 and 109.

Based on these confirmed improvements, weekly sessions of ultrasound-guided nerve mobilization were continued alongside the existing physical therapy. Throughout this mobilization phase, the patient remained pain-free (NRS 0/10) while motor function progressively improved. By day 109, finger and thumb extension strengths improved substantially, and grip strength approached that of the contralateral side (Table 1; Figures 2C,D). Serial ultrasound examinations demonstrated progressive shrinkage of the ganglion to 37.13 mm^2^. The PIN showed an initial increase in CSA to approximately 3.54 mm^2^, suggesting release from focal flattening, before stabilizing at 2.51 mm^2^ with a more rounded contour and clearer fascicular pattern (Figures 3A,B). Dynamic short-axis ultrasonography confirmed improved local sliding between the PIN and the overlying soft tissues. The acute increase in sliding velocity observed in the exploratory PIV analysis was highly consistent with these subsequent qualitative improvements in nerve excursion observed throughout the follow-up period.

## 4 Discussion

We report the successful treatment of ganglion-associated PIN palsy using ultrasound-guided aspiration and nerve mobilization. While aspiration relieved pain, motor deficits persisted, implying decompression alone was insufficient. Subsequent nerve mobilization yielded substantial recovery, concurrent with morphological normalization. Although surgery is standard for ganglion-associated PIN palsy (14–16), conservative approaches (immobilization, steroid injection, spontaneous resolution) can be effective (13, 17, 18). In the present case, aspiration and physical therapy reduced pain and ganglion size, yet motor deficits persisted. The temporal pattern of improvement in this case provides insight into the potential mechanisms underlying the effect of ultrasound-guided nerve mobilization, as illustrated by the longitudinal changes in nerve CSA and clinical measures (Figure 2, Table 1). During the aspiration phase, dorsal forearm pain decreased from an NRS score of 4/10 to 1/10, and the ganglion CSA was reduced from 67.06 to 58.96 mm^2^; however, finger and thumb extension remained at manual muscle testing grade 1, and finger extension in daily activities was still impossible. Furthermore, dynamic ultrasound demonstrated persistent flattening of the PIN at the compression site. Crucially, although the ganglion CSA significantly decreased during this 4-week aspiration phase, the nerve CSA remained flattened and unchanged at approximately 1.70 mm^2^. Morphological restoration—characterized by a transient “rebound” increase in CSA (Day 44)—occurred only after the initiation of mobilization. This discrepancy effectively rejects the hypothesis that the observed recovery was merely a delayed effect of the initial aspiration. Instead, it indicates that reducing extrinsic pressure alone was insufficient to release the nerve from its entrapped state, and that mechanical mobilization was necessary to overcome the intraneural or extraneural tethering that maintained the focal compression. This persistent entrapment, despite the reduction in extrinsic pressure from the ganglion, suggests that chronic compression may have induced micro-adhesions or fibrosis between the nerve epineurium and the surrounding fibro-osseous structures within the radial tunnel. In such cases, the nerve remains “tethered” even after the initial space-occupying lesion is addressed. Mechanical mobilization likely facilitated the release of these perineural adhesions and improved the compliance of the surrounding soft tissues, such as the brachioradialis and supinator fascia. This process potentially restored the intraneural microcirculation and axoplasmic flow, which are essential for axonal regeneration and the recovery of motor unit recruitment in the PIN-innervated extensors. Therefore, we suggest that true functional decompression required not only the reduction of extrinsic pressure via aspiration but also the restoration of nerve compliance and gliding through mechanical mobilization.

After ultrasound-guided nerve mobilization was introduced, marked local sliding between the PIN and the overlying brachioradialis muscle became apparent under real-time short-axis ultrasound in the frontal plane, clinical motor recovery accelerated, finger and thumb extension strength increased from grade 2 to grade 4, and grip strength on the affected side approached that of the contralateral hand. The parallel improvement of nerve–soft tissue sliding on ultrasound and functional gains support the hypothesis that restoration of nerve–soft tissue sliding is an important therapeutic target in this condition, consistent with the serial morphological changes observed on short-axis ultrasound (Figure 3). From neurophysiological and biomechanical perspectives, several mechanisms may contribute to the effects of nerve mobilization in entrapment neuropathies. Previous experimental and clinical studies have suggested that nerve-gliding techniques can reduce intraneural and extraneural adhesions, improve perineural microcirculation, and modulate intraneural pressure, thereby facilitating axonal transport and nerve conduction (13, 17–22). In the present case, ultrasound-guided nerve mobilization was performed with the elbow flexed and the forearm in supination, while gentle manual mobilization was applied over the brachioradialis region to promote the sliding of the PIN around the ganglion and adjacent tissues. Visual confirmation of nerve motion on ultrasound may have helped the therapist titrate the intensity and direction of mobilization, minimizing excessive strain on the nerve while focusing on restoring local sliding (23–25). The observed changes in nerve CSA—from a flattened configuration at the compression site to a more rounded contour with a clearer fascicular pattern—together with improved local sliding between the PIN and the overlying soft tissues on short-axis scanning in the frontal plane, were consistent with decreased focal compression and an improved mechanical environment of the nerve (8, 21). These biomechanical changes likely contributed to the delayed but substantial motor recovery observed after initiation of nerve mobilization (26–28).

The PIV analysis provided objective evidence that ultrasound-guided nerve mobilization significantly improved the transverse tissue gliding around the posterior interosseous nerve (p < 0.05). While most previous reports rely on qualitative descriptions (29–32), our quantitative data aligned with the clinical recovery, supporting its potential utility despite being limited to a single within-session comparison. Furthermore, the fact that this improvement in gliding was accompanied by a lack of increased pain (NRS 0) effectively rejects the possibility that the transient increase in nerve CSA on Day 44 represented reactive edema or inflammation caused by the manual intervention. Instead, this transient enlargement likely represents the “unmasking” of intraneural edema following the release of long-standing mechanical stress. Previous studies suggest that chronic compression can cause intraneural edema, which may be obscured by focal flattening under high pressure (21, 22). In this case, the rounded nerve profile and clearer fascicular pattern observed on Day 44 further support the interpretation that mobilization facilitated the restoration of the nerve's internal environment. Although this analysis was limited to a single within-session comparison, and the metric has not yet been validated as a clinical outcome measure, the concordance between the PIV-derived increase in sliding velocity and qualitative ultrasound observations supports the feasibility of using PIV to objectively capture mechanical changes induced by nerve mobilization *in vivo*.

Our study has several limitations inherent to a single-case report. First, the absence of a control group and blinding means we cannot completely rule out the placebo effect or observer bias, particularly regarding subjective measures such as MMT. Future studies should incorporate more objective strength measurements, such as dynamometry for finger extension, or standardized functional performance tests to strengthen outcome assessments. Second, although we argue that mobilization provided the critical mechanical release, it is physiologically possible that the initial aspiration initiated a slow recovery process that overlapped with our intervention. However, the distinct temporal correlation between the mobilization intervention and the objective morphological changes in the nerve (CSA rebound and rounding) strongly supports the efficacy of the manual technique. Finally, while PIV analysis offers an objective, computational metric for nerve gliding—minimizing observer bias compared to visual estimation—it remains an exploratory method requiring further validation. Nevertheless, the high intra-rater reliability of our ultrasound measurements (ICC 0.88–0.93) further strengthens the validity of the observed morphological changes. While the placebo effect can never be entirely excluded in a case report, the synchronized improvement in objective biomechanical markers (PIV and CSA) and clinical outcomes provides a compelling rationale for the role of mobilization.

Regarding patient selection criteria, this conservative stepwise approach may be particularly indicated for patients who present with purely motor deficits without severe acute axonal loss, and in whom high-resolution ultrasonography confirms an anatomically intact nerve compressed by a reducible soft-tissue mass. Conversely, patients with rapid progression of profound weakness, long-standing fixed deformities, or space-occupying lesions unresponsive to initial aspiration may be better candidates for early surgical decompression. Careful baseline evaluation and dynamic ultrasound monitoring are essential to identify appropriate candidates for this conservative strategy.

Despite these limitations, this case illustrates that a stepwise conservative strategy combining ultrasound-guided aspiration and nerve mobilization can lead to motor recovery in ganglion-associated PIN palsy, potentially averting surgery. Dynamic ultrasonography serves as a valuable tool for guiding mobilization and objectively monitoring nerve morphology and excursion. Future studies should focus on identifying optimal candidates for this approach and developing validated quantitative metrics for nerve sliding to establish its clinical utility. Furthermore, as acknowledged, the absence of electrodiagnostic studies limits the definitive confirmation of baseline nerve severity. Including nerve conduction studies and electromyography in future cases will enhance diagnostic rigor and allow better comparison with the existing literature.

## 5 Patient perspective

When I noticed that I could no longer extend my fingers or thumb, I became anxious. I rely on my right hand in my daily work and at home, and I was worried that I might need surgery and that my hand might never fully recover. During my visits, my clinicians showed me ultrasound images and carefully explained what the ganglion and nerve looked like and that we could first try aspiration and a rehabilitation program instead of going straight to surgery.

Initially, the pain improved, but the weakness in my fingers did not change significantly and I sometimes felt frustrated. However, being able to see on the ultrasound that the cyst was shrinking and that the nerve was moving more freely helped me stay motivated to continue with the nerve mobilization and exercises. I am very grateful that my finger extension and grip strength gradually improved without surgery and that I was involved in deciding on each step of my treatment.

## Data Availability

The original contributions presented in the study are included in the article/Supplementary Material, further inquiries can be directed to the corresponding author.
