# Supplementary material for: Case Report: Ultrasound-guided nerve mobilization and rehabilitation for non-traumatic posterior interosseous nerve palsy associated with a ganglion cyst
Source: Front Rehabil Sci. 2026 May 20;7:1811498. doi: 10.3389/fresc.2026.1811498 (PMC13229876; doi:10.3389/fresc.2026.1811498)
Supplement: Supplementary file 1 [file Datasheet1.pdf]

CARE Checklist of information to include when writing a case report

CARE case report guidelines

BY-NC-ND

Topic	Item No	Checklist item description	Reported on Page Number/Line Number	Reported on Section/Paragraph
Title	1	The diagnosis or intervention of primary focus followed by the words "case report"	Page 1, Line 1	
Key Words	2	2 to 5 key words that identify diagnoses or interventions in this case report, including "case report"	Page 1, Line 18	
Abstract (Structured summary)	3a	Background: state what is known and unknown; why the case report is unique and what it adds to existing literature.	Page 2, Line 27	
	3b	Case Description: describe the patient's demographic details, main symptoms, history, important clinical findings, the main diagnosis, interventions, outcomes and follow-ups.	Page 2, Line 30	
	3c	Conclusions: summarize the main take-away lesson, clinical impact and potential implications.	Page 2, Line 42	
Introduction	4	One or two paragraphs summarizing why this case is unique **(may include references)**	Page 2-3, Line 59-71	
Patient Information	5a	De-identified patient specific information	Page 3, Line 73	
	5b	Primary concerns and symptoms of the patient	Page 3, Line 73-75	
	5c	Medical, family, and psycho-social history including relevant genetic information	Page 3, Line 74	
	5d	Relevant past interventions with outcomes	N/A	
Clinical Findings	6	Describe significant physical examination (PE) and important clinical findings	Page 3, Line 76-82	
Timeline	7	Historical and current information from this episode of care organized as a timeline	Page 4, Line 124	
Diagnostic Assessment	8a	Diagnostic testing (such as PE, laboratory testing, imaging, surveys).	Page 3, Line 98-99	
	8b	Diagnostic challenges (such as access to testing, financial, or cultural)	Page 3, Line 93-95	
	8c	Diagnosis (including other diagnoses considered)	Page 3, Line 95-98	
	8d	Prognosis (such as staging in oncology) where applicable	N/A	
Therapeutic Intervention	9a	Types of therapeutic intervention (such as pharmacologic, surgical, preventive, self-care)	Page 3, Line 108-110	
	9b	Administration of therapeutic intervention (such as dosage, strength, duration)	Page 3-4, Line 111-115	
	9c	Changes in therapeutic intervention (with rationale)	Page 5, Line 135-136	

Follow-up and Outcomes	10a	Clinician and patient-assessed outcomes (if available)	Page 5, Line 149-151	
	10b	Important follow-up diagnostic and other test results	Page 5, Line 151-154	
	10c	Intervention adherence and tolerability (How was this assessed?)	Page 5, Line 148-149	
	10d	Adverse and unanticipated events	Page 6, Line 218-219	
Discussion	11a	A scientific discussion of the strengths AND limitations associated with this case report	Page 7, Line 230-238	
	11b	Discussion of the relevant medical literature **with references**	Page 6, Line 199-202	
	11c	The scientific rationale for any conclusions (including assessment of possible causes)	Page 6, Line 183-187	
	11d	The primary "take-away" lessons of this case report (without references) in a one paragraph conclusion	Page 7, Line 244-246	
Patient Perspective	12	The patient should share their perspective in one to two paragraphs on the treatment(s) they received	Page 7, Line 251-260	
Informed Consent	13	Did the patient give informed consent? Please provide if requested	**Yes** ☑ Page 7, Line 265	**No** ☐

Please leave this space alone as it will be supplemented by the editorial office when needed.
